# The meaning of mass extinctions and what the fossil record tells us about angiosperm survival at K-Pg: a reply to Hagen (2024)

**DOI:** 10.1098/rsbl.2024.0265

**Published:** 2024-08-28

**Authors:** Jamie Thompson, Santiago Ramírez-Barahona

**Affiliations:** ^1^ School of Biological Sciences, University of Reading, Whiteknights, Reading, Berkshire, UK; ^2^ The Milner Centre for Evolution, Department of Life Sciences, University of Bath, Bath, UK; ^3^ Departamento de Botánica, Instituto de Biología, Universidad Nacional Autónoma de México, Ciudad de México, México

**Keywords:** mass extinction, K-Pg, angiosperms, phylogenetics, diversification, extinction

## Abstract

Last year, we published research using phylogenetic comparative methods (PCMs) to reveal no phylogenetic evidence for elevated lineage-level extinction rates in angiosperms across K-Pg (Thompson JB, Ramírez-Barahona S. 2023 No phylogenetic evidence for angiosperm mass extinction at the Cretaceous–Palaeogene (K-Pg) boundary. *Biol. Lett.* **19**, 20230314. (https://doi.org/10.1098/rsbl.2023.0314)), results that are in step with the global angiosperm fossil record. In a critique of our paper (Hagen ER. 2024 A critique of Thompson and Ramírez-Barahona (2023) or: how I learned to stop worrying and love the fossil record. *Biol. Lett.* **20**, 2020240039 (https://doi.org/10.1098/rsbl.2024.0039)), simulation work is presented to argue we erred in our methodological choices and interpretations, and that we should have deferred to fossil evidence. In our opinion, underlying this critique are poor methodological choices on simulations and philosophical problems surrounding the definition of a mass extinction event, which leads to incorrect interpretations of both the fossil record and PCMs. We further argue that deferring to one source of evidence in favour of the other shuts the door to important evolutionary and philosophical questions.

## 1. The meaning of mass extinctions

The main contention of the critique [1] is that our findings disagree with the angiosperm fossil record [2–4]. The discrepancies between PCMs and fossils are partly what motivated our work, but we disagree that regional fossil records should be trusted over molecular clock evidence (or vice versa). As practitioners of molecular clock analyses, we are well-versed in the issues surrounding PCMs, but we are also aware of the taxonomic, temporal and spatial uncertainties underlying the fossil record. With this in mind, to us, the important philosophical question is not to corroborate fossils with phylogenies or defer to one in favour of the other. Rather, as previous work has been done [5–8], we should ask when and why PCM methods and data tell different stories than those revealed by fossils. Only through these questions can we aim towards the integration of different sources of evidence, and their associated uncertainties, biases and dimensionalities, into a mature and comprehensive interpretation of macroevolution.

More often than not, debates weighing PCMs against fossils fall back to stating that the evolutionary patterns gleaned from analyses of molecular phylogenies, as opposed to those of the fossil record sometimes differ widely [1], but without asking why this happens. Fossils are the only concrete evidence of the past, but this evidence is limited and comes with high spatial and taxonomic biases and uncertainties. These are two crucial aspects that often go unmentioned [1], but that lead to important compatibility problems between phylogenetic and fossil evidence that warrant consideration. From the onset, Hagen states we argue there is no evidence that angiosperms were ‘affected’ by the Cretaceous–Palaeogene (K-Pg) mass extinction. We agree this argument is highly flawed and not true, but these were never our words [9]. In fact, we originally concurred that ‘the K-Pg extinction event caused widespread plant species-level extinctions and changes in ecosystem composition at local and regional scales’ [9].

Here lies a problem on the meaning with ‘mass extinctions’ and a critical confusion in reading the fossil record, that between regional species extirpation and true global extinction of lineages. High levels of species extinction cannot be the sole metric to measure the macroevolutionary impacts of a mass extinction event, more so when data come from specific local assemblages. Defining a mass extinction event solely based on high rates of species-level extinction begs the question whether PCMs are futile attempts to study the effects of the K-Pg event on angiosperms (or by the same standards any event on any other group). As we originally discussed, with deep-time phylogenies, we can only search for signatures of extinction at higher taxonomic ranks (lineages). Thus, a more appropriate definition of a mass extinction—the one we originally adopted [9]—entails a geographically widespread increase in extinction suffered by higher taxa (genera, families, orders), accompanied by a temporary decline in their standing species diversity [10].

## 2. What the fossil record tells us about angiosperm survival at K-Pg

Ample fossil evidence reveals the remarkable survival of angiosperm lineages (e.g. families, orders) in the face of substantial species-level extinction rates across the K-Pg [11–14]. This is a major point underlying our original argument that despite high rates of species-level extinction, few to no living angiosperm families (or orders) were lost globally [13]. These patterns can even be observed at the locations from the Northern hemisphere that show the clearest signals of massive species extinctions [11], in which the less diverse Palaeogene floras still contain many of the higher taxonomic lineages observed prior to the K-Pg. By brushing off this evidence and ignoring critical geographical and taxonomic features of mass extinctions, the fossil record is not given the credence the critique is pleading for.

The North American leaf macrofossil record (e.g. Hell Creek flora) shows extinction dynamics that are taken to disagree with our results [2,3,15]. Indeed, leaf macrofossils do conservatively support a scenario of 57% species extinction across North America [3,13], but the palynoflora suggests a minimum of 30% extinction [3]. This is expected because macrofossils often sample the flora at a higher taxonomic resolution than pollen [16]. These, often unseen, differences in taxonomic resolution extend into molecular phylogenies, which sample past diversity at far lower taxonomic resolution than the fossil record. However, the fact there is little to no phylogenetic evidence of disappearing angiosperms lineages at K-Pg is not merely a problem of resolution, but an observation that in fact agrees with the fossil record.

While the North American record may be representative of the overall pattern of species-level extinctions—fossil dynamics support species-level extinctions across the globe—extrapolating this across the planet is not straightforward due to spatial and taxonomic heterogeneity [13]. That sites further from the Chicxulub impact site show a ‘muted’ K-Pg extinction has been discussed previously [17]. Possibly the most divergent example is the pollen record from La Colonia formation (Argentina) [18] where only 12% of local angiosperm taxa disappeared at K-Pg, providing regionally consistent evidence of angiosperm survival across the boundary [19]. Furthermore, the few families that disappeared at La Colonia survived elsewhere to the current day, indicating regional extirpation of major lineages instead of their global disappearance.

**Figure 1.**
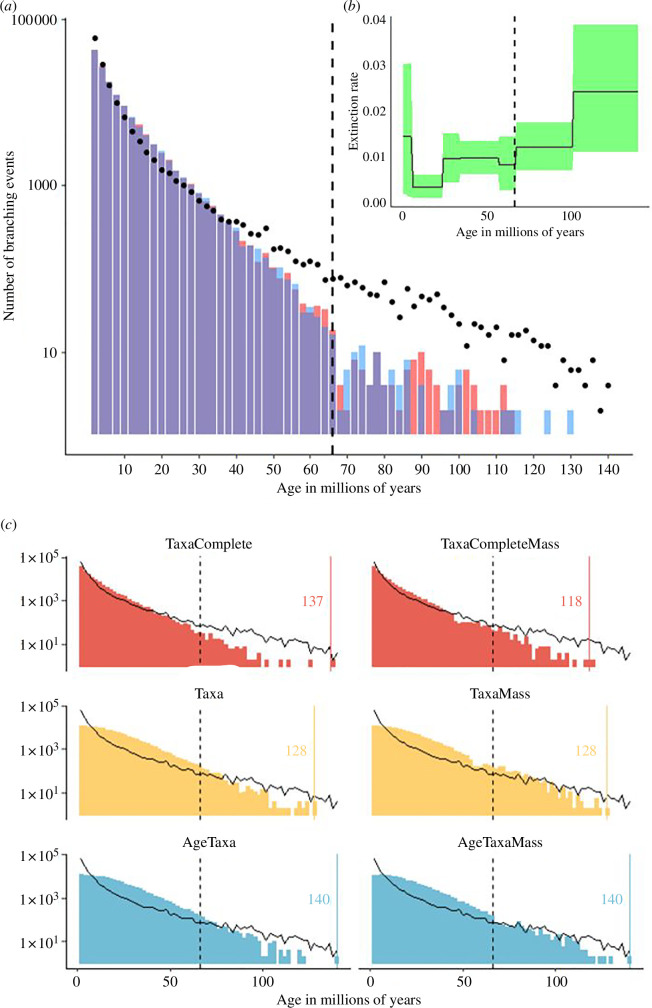
Inconsistencies between simulated phylogenies and the angiosperm molecular phylogeny, fossil-estimated genus-level extinction rates across the K-Pg boundary and impacts of parameter choices on simulated phylogenies. (*a*) Decoupled lineage accumulation rates in the molecular phylogeny produced by Smith & Brown [21] (black dots) and simulated phylogenies by Hagen [1] (red bars = 10% survival at K-Pg, blue bars = 40% survival at K-Pg). (*b*) Global genus-level extinction rates of angiosperms, with no support for acceleration across the K-Pg, reproduced from Silvestro *et al.* [20]. (*c*) Variation in lineage accumulation rates and root ages by different combinations of parameter choices (‘Age’ = conditioned on root age, ‘Complete’ = conditioned assuming all living known species are sampled, ‘Mass’ = simulated conditioned with a simulated mass extinction, ‘Taxa’ = conditioned on number of species sampled). In all plots, K-Pg is indicated with a vertical dashed line and coloured vertical lines mark the simulated tree height in millions of years.

The strongest support for evolutionary dynamics will always come from fossils [1]. Indeed, at higher taxonomic levels—genus and family—angiosperm survival is supported through analysis of the global macrofossil record [20], and no significant change in extinction rates across K-Pg is recovered (figure 1*b*). A 60−70% loss of higher lineage diversity at K-Pg is unrealistic and at odds with the global fossil record, especially for higher taxonomic lineages [13,20].

## 3. The reliability of inferences from phylogenies

Remarkable angiosperm lineage survival across K-Pg is strongly supported by robust phylogenetic divergence estimates [21–24], including a recent comprehensive phylogenomic reconstruction sampling of almost 8000 living genera [25]. Here, estimated stem ages for 378 families predate K-Pg, which are not simply the result of being spuriously pushed into the deeper past by the molecular clock—fossils for numerous families have a minimum age of 66 Ma or older [21,23]. There is undoubtedly a hidden diversity of Cretaceous angiosperm families that went extinct at K-Pg, but most likely these represent but a minor fraction that is far from the 60−90% of Cretaceous angiosperm higher-level diversity required by the critique’s simulations.

Hagen begins by replicating our model selection analysis, finding the same result we did, that constant-rate birth–death models are ‘astronomically’ better than episodic or mass extinction models [1,9]. However, accurate replication requires careful analysis. We highlight issues with the code used in the replication of our results that reduce the confidence ([26] V1), such as pruning the three orders of the ANA grade (these are important both phylogenetically and because of their excellent fossil record [27–29]), accidentally pruning approximately 1700 more tips (approx. 2% of tips in the tree), and using an old version of the Smith & Brown tree [21,22] that is not corrected for duplicate species, infraspecific taxa or taxonomic issues [30].

While we are aware that estimating extinction rates from extant-only phylogenies is contentious [31], simulations demonstrate it is possible with large phylogenies [32] and when extinction events entail low survival rates [33], i.e. when hyperdiverse lineages suffer massive extinctions. Indeed, the same simulations taken to disapprove our main conclusions support our original goals and justify our methodological choices: if angiosperms in fact suffered a 70−90% mass extinction of lineages—not species—at K-Pg, our methods would be robust enough to detect it [34]. Testing the reliability of methods and their sensitivity under varying realistic conditions is crucial for any analysis using PCMs. To this point, Hagen provides a set of simulations in an attempt to demonstrate that our methodological choices were inadequate, but upon closer inspection, we have concerns about the simulation’s methodology.

To address the incapacity of CoMET [34] to identify mass extinctions in the rootward half of the tree, due to the decrease in inferable information with temporal depth from the present day, Hagen produced and analysed five simulated trees intended to be comparable to empirical trees. These simulations are only slightly justified when considering only one of the two empirical trees we used (the Smith & Brown tree [21]), whose ages are at the lower extreme of estimations [5–7,25]. Here, K-Pg occurs approximately 47% towards the root, whereas in the Zanne *et al*. tree [22] it is approximately 27%. It must be noted that the Smith & Brown tree adheres to the ideal properties originally described by the authors of CoMET [34], in which ‘a candidate study tree should ideally be approximately 2−3 times the age of the putative mass-extinction event, but the event should not occur in the last approximately 15% of the tree height’. In any case, CoMET successfully detected simulated mass extinctions with 10−20% lineage survival, which is what we would expect—albeit erroneously—if species-level extinction rates mirror elevated extinction of entire lineages. Also, despite K-Pg being in the rootward half of one simulated tree (approx. 58% in the 114 million-year-old tree), CoMET still successfully detects a mass extinction under these non-ideal conditions. As expected, the signal for simulated extinctions disappears when lineage survivorship is higher (30−40%), and it is this detection failure that Hagen takes as proof of the method’s inadequacy. We agree that CoMET is limited when it comes to weaker (non-mass) extinction events, but that is not what the method was intended to do.

Similarly, the test for the ‘Grand Coupure’ extinction event (33.5 Mya) is intended to simulate trees with ‘a K-Pg that 10% survive and a Grand Coupure event that 80% survive’ [1]. Hagen argues that in this scenario CoMET misses K-Pg and only detects a change in extinction rates at 33.5 Mya, proving the method’s inadequacy in more complex macroevolutionary scenarios. However, we have noticed that survival fractions are switched in Hagen’s code: survival fraction of 0.1 for the Grand Coupure and 0.8 for the K-Pg ([26] V1). Considering the two survival fractions coded into simulations, these analyses instead support CoMET’s ability to detect mass extinctions, albeit in this case the simulated Grand Coupure mass extinction (interestingly, this event occurs very close to the last approximately 15% of the tree height, where CoMET’s power to detect mass extinction diminishes [33]).

Phylogenies should be simulated in a way that ensures replication and parameter control, aspects that are crucial when identifying factors affecting sensitivity of methods. We appreciate the difficulty encountered when simulating trees to test the efficacy of a PCM, but are concerned with the method employed. We identified several areas of methodological concern that reduce confidence when interpreting Hagen’s simulations (figure 1*a* and *c*). These trees were simulated with episodic branching models, in which speciation and extinction rates differ before and after K-Pg, but without any clear justification for this model or its parameters. This is a curious methodological choice given that constant rate models are ‘astronomically’ better supported in both our original paper and the replication of our results in Mesangiospermae [1,9]. More critically, Hagen’s simulations incorrectly assume a complete sampling of living species, instead of a sampling fraction of approximately 20–25%. Simulations do not condition on age, only on the number of taxa (70 000 species), thus the distribution of branching times, root height and age of living lineages are not comparable (figure 1*a*), even among simulations. We found great variability in branching times and root height among simulated trees, the latter varying from approximately 114 to approximately 143 million years (the simulated tree for the ‘Grand Coupure’ analysis has a root height of approx. 209 million years). Some of these angiosperm crown ages are unrealistic [5–7,25], but this crucial aspect of the simulations goes unmentioned.

An alternative way to perform simulations, that may be more appropriate for comparison with angiosperm evolution, might be to condition both on age and number of taxa [35]. To show the impacts of these methodological choices on tree shape, we constructed simulated trees conditioning both on age and number of taxa (*n* = 70 000, age = 140) and compared to trees conditioned only on age. We found great differences among trees associated with parameter choices (figure 1*c*), where root height and the distribution of internal node ages varied as a function of several simulation parameters, except when simultaneously conditioning on age and number of taxa. Our simulations are only aimed at proving that the overall shape of trees is highly sensitive to parameter settings, thereby affecting inferences and comparability. Finally, the total number of sampled MCMC iterations in Hagen’s analyses (10 000 sampled every 100), done on five simulated trees (one per analysis), reveal convergence issues. Typically, we would expect Bayesian CoMET analyses to be run for millions of iterations, to leave the ‘burn-in’ phase and reach effective sample sizes of at least 200 for relevant parameters, indicating stationarity [36–39].

## 4. Embracing the gap between phylogenies and fossils

In the critique, Hagen argues that the fossil record should be trusted over phylogenies. We did not once promote the alternative, that phylogenies should be given more credence than fossils. The crux of the matter is that of understanding the fossil record and PCMs rather than blindly trusting or loving either one. We agree further research is needed, but taking the default stance and deferring to the fossil record over PCMs (or *vice versa*) is detrimental to this goal. Failing to fully acknowledge the potential of integrating fossils and phylogenies inevitably leads to the need to ‘corroborate’ or ‘defer’ to one source of evidence in favour of the other. By uncritically taking sides in this false dichotomy, important evolutionary questions and philosophical matters remain out of reach, not only about the reliability of different methods and data to reconstruct the distant past but also about the way we observe and think about the distant past. As previous works have been done, embracing the uncertainties and limits of both PCMs and the fossil record has the potential to lead to key insights not only for our understanding of plant macroevolution [8,40–42], but also on the ecological and evolutionary processes that have shaped the diversity of life we see today.

## Data Availability

Our code is made available at Zenodo [43]. Supplementary material is available online [44].
